# So You Want Your Child to Be a Genius?

**DOI:** 10.1371/journal.pbio.0040139

**Published:** 2006-05-16

**Authors:** Jonathan Flint

## Abstract

Geneticist Jonathan Flint reviews David Plotz's book *The Genius Factory: The Curious History of the Nobel Prize Sperm Bank.*

For many, it seems obvious that some people are simply smarter than others, that intelligence is a unitary trait, and that individual differences are accurately reflected in the intelligence quotient (IQ) obtained from an intelligence test. Others, while accepting that intelligence exists, assert that it is far too subtle a concept to be encapsulated in a single number. Howard Gardner proposed that there are half a dozen different intelligences that are independent of one another [ 1]. Recourse to the data to decide who's right only serves to sharpen the debate, as protagonists take positions on the relative merits of such mathematical complexities as factor analysis, principal components, and orthogonal rotations. Stephen J. Gould has a nice pictorial demonstration that the extraction of a general factor representing unitary intelligence, the so-called g-factor, from a set of correlated IQ test scores is, as he sees it, a specious exercise with infinitely many solutions [ 2].

The inability to settle on a measure of intelligence makes exploring its relationship to genetics even more complicated. It's possible to find those who argue against the existence of IQ, against the heritability of IQ, and, going a little further, against the value of quantitative genetic analyses of how genetic variation contributes to human behaviour. Nevertheless, psychologists still work with intelligence tests, using separate measures of performance and verbal IQ, as well as the full-scale IQ score (the one that needs to be over 150 if you want to become a MENSA member), largely because there isn't anything better. There are certainly circumstances when you need these measures, clinically speaking. I've seen children referred for elective mutism (hard to believe anyone would complain about a ten-year-old keeping quiet) who, on testing, turned out to be learning disabled.

Some of these children owe their disability to a genetic mutation. If you plot the IQ distribution in the population, the well known bell shaped curve (with the majority of people having a mid-range IQ and fewer numbers of people having very high or very low IQs) is interrupted at the lower end of the distribution by a small blip. This has long been attributed to the combined effects of chromosomal abnormalities (predominantly Down syndrome) and single gene mutations, such as the Fragile X syndrome. There's not so much dispute about the importance of genetic effects on moderate to severe learning disability. Instead, arguments have raged over the contribution of genetic variants to the main bulk of the curve, though nowadays the heat has largely gone out of the debate. In fact, last year the first genome-wide analysis with respect to IQ was reported, in which researchers in Holland and Australia showed a significant linkage for performance IQ to a locus on Chromosome 2 [ 3].

I wouldn't like to suggest that molecular genetics will be the final arbiter in the argument over genetic effects on intelligence: it can hardly be said to have clarified the biology, or lack of it, in other complex phenotypes. Just like the question of the value and meaning of IQ tests, data by themselves may not be enough to convince sceptics. The primary tool used to address whether genes influence intelligence, the twin study (in which commonalities are examined between genetically identical twins), has generated technical discussions of such formidable complexity that it sometimes appears as if both sides of the argument can claim victory.

There are alternative strategies to the twin study that can be used to assess genetic effects on intelligence. One that does not get much of a mention is artificial insemination. Artificial insemination makes it possible for one male to have thousands of offspring, all of which derive half their genetic constitution from the same father. Since each child would be brought up in a different environment, by a different mother, the genetic effect on IQ could be estimated by looking for correlations in the offsprings' IQ. If that sounds like an unlikely experiment, think again. It's already been done.

Unfortunately, this unwitting experiment doesn't add much to the IQ genetics debate, but it's an interesting story, well told by David Plotz. Artificial insemination was used for the same reasons it is adopted by farmers: to maintain, or improve, the fitness of their stock. Here's a successful example: Doron (Greek for “gift”) Blake used a computer at age two, played chess when he was five, learned algebra in kindergarten, and wrote his first book a year later. His mother, “transpersonal psychologist” Afton Blake, really wanted to make sure she had an outstanding offspring. Not by playing Mozart to the foetus, not by adherence to a strict organic diet during pregnancy (supplemented by vitamins), not by paying for the best education, nor even by home tutoring. Instead, she applied to Robert Graham's Repository for Germinal Choice, the Nobel sperm bank.

The Nobel laureate Hermann Muller had convinced Graham that a build-up of atmospheric radiation was damaging humanity's DNA, condemning the world to a genetic decline that could be reversed only by freezing the sperm of the world's best men and using it to breed future generations. From the 1970s, Graham, an optometrist who created shatterproof plastic lenses, had been collecting the sperm that would be distributed, for free, to women of the right quality. The Nobel Prize sperm bank would populate the world with brilliant people, like Doron, the outstandingly successful result of an insemination by Donor Red #28.

Actually, Donor Red #28 wasn't a Nobel Prize winner. Only three laureates contributed, and there are no children from any of them. The small quantity of Nobel sperm had been sent out within the first year of the bank's opening, and no pregnancies occurred (no in vitro fertilisation was used; we're talking teaspoons here). After that, there were no more Nobel donors, primarily because one of the three Nobelist contributors went public: William Shockley, co-inventor of the transistor, founding father of Silicon Valley (which is good), believer in eugenics (which is bad), who opined, “Just as the autobahns were a good thing, maybe there were some other good things about Hitler.” Shockley was the sort of man who, when he won a libel suit against a journalist for comparing him to Hitler, was awarded $1 in damages. Support from Shockley was enough to discredit Graham's bank and dissuade his fellow Nobelists from contributing.

Without Nobel Prize sperm you might think the Nobel Prize sperm bank wouldn't do so well, but by the time it closed there were 215 children. Whose kids were they? If not Nobelists, maybe almost Nobelists, like Jonas Salk? At least 30 children are the offspring of Donor Coral #36, described in the Repository's report as “a professional man of very high standing in his field, has had a book published, excels in mathematics … very good looking, happy, slightly extrovert, very easy going … excels in many sports.” David Plotz tracked down Donor Coral #36 (one of the things you learn from this book is that there is no such thing as anonymity in sperm donation) and discovered that “Jeremy,” as we come to know him, is not quite as advertised. He's supposed to have an IQ of 160, but he never took an IQ test (“I told them the number I thought they would want to hear”). Jeremy does not live in Salk's neighbourhood. “Jonas Salk would have been afraid to even drive through here … The house was a wreck. Windows were boarded up with plywood; gutters drooped; siding was dangling off.” (Fortunately, it turns out Plotz has the wrong house. Jeremy's house is next door; he just happens to live next to some drug dealers). Jeremy isn't even near Nobel status. Nor, it seems, are the other donors. There are a couple of university professors, a self-made businessman (no, not a drug dealer), a former math prodigy, a sculptor, a political activist (“I recognized his name from news stories about his repellent ideas”), and “Michael,” son of a Nobelist. This sounds more hopeful, and Michael has contributed a lot of sperm. But it seems that the only thing Michael has produced in his life is sperm. Michael has spent fifteen years of his life masturbating. “It had, he admitted, been exhausting.”

Given the quality of this crowd of donors, it's no surprise that Plotz comes to doubt the value of genius sperm. He blames success on the mum. Give them the sperm from the dumbest player on the NFL's worst team, and they'll still turn out a brilliant boy, like Doron. Plotz finds the ex-child prodigy in his freshman year at Reed College, intending to major in comparative religion and then get a job teaching at his old high school. This is not quite what Graham had in mind. Even though Doron is smart enough to charge the press for his time (he did more than 100 interviews in 2001; at a rate of $500 or more, not such a bad income), he's not the genius inventor of Graham's imaginings. But then would you really want a genius for a child? Shockley, for instance?

The history of the Nobel sperm bank is a story of the American bizarre, a reminder of what that culture could accommodate. Graham, before he started the sperm bank, tried to start a country, Grahamland. He sent the estate agents looking for an island suitable for establishing an elite research colony (there were a few islands in the Atlantic that the United Kingdom might part with), whose inhabitants would enjoy the best laboratory facilities and could devote their lives to inventing. Muller, a committed socialist who had to flee Stalinist Russia, ends up a professor at the University of Indiana. Makes you think. Would that be allowed under the current regime?

**Figure:**
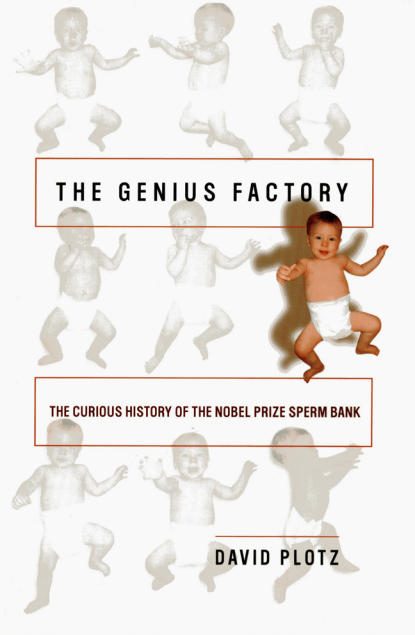


## Abbreviation

IQ: intelligence quotient
